# Are synchronous chats a silver lining of emergency remote instruction? Text-based chatting is disproportionately favored by women in a non-majors introductory biology course

**DOI:** 10.1371/journal.pone.0273301

**Published:** 2022-10-19

**Authors:** Rachael D. Robnett, Cissy J. Ballen, Sheritta Fagbodun, Kelly Lane, Sophie J. McCoy, Lecia Robinson, Ebony I. Weems, Sehoya Cotner

**Affiliations:** 1 Department of Psychology, University of Nevada Las Vegas, Las Vegas, NV, United States of America; 2 Department of Biological Sciences, Auburn University, Auburn, AL, United States of America; 3 Department of Biology, Tuskegee University, Tuskegee, AL, United States of America; 4 Department of Biology Teaching and Learning, University of Minnesota, Minneapolis, MN, United States of America; 5 Department of Biological Science, Florida State University, Tallahassee, FL, United States of America; 6 Department of Biological and Environmental Sciences, Alabama Agricultural and Mechanical University, Huntsville, AL, United States of America; 7 Department of Biological Sciences, University of Bergen (Norway), Bergen, Norway; 8 Department of Biology Teaching and Learning, University of Minnesota, Minneapolis, MN, United States of America; Dai Hoc Duy Tan, VIET NAM

## Abstract

The coronavirus disease 2019 (COVID-19) pandemic has led to a reimagining of many aspects of higher education, including how instructors interact with their students and how they encourage student participation. Text-based chatting during synchronous remote instruction is a simple form of student-student and student-instructor interaction. The importance of student participation has been documented, as have clear disparities in participation between those well-represented and those under-represented in science disciplines. Thus, we conducted an investigation into who is texting, what students are texting, and how these texts align with course content. We focused on two sections of a large-enrollment, introductory biology class offered remotely during Fall 2020. Using an analysis of in-class chatting, in combination with student survey responses, we find that text-based chatting suggests not only a high level of student engagement, but a type of participation that is disproportionately favored by women. Given the multiple lines of evidence indicating that women typically under-participate in their science courses, any vehicle that counters this trend merits further exploration. We conclude with suggestions for further research, and ideas for carrying forward text-based chatting in the post-COVID-19, in-person classroom.

## Introduction

The coronavirus disease 2019 (COVID-19) pandemic has presented college instructors with a defining moment in higher education, as most have been called to fundamentally restructure our courses and interactions with students during an emergency shift to remote instruction [1, 2]. In so doing, instructors have had to meaningfully reconsider their curricula—what learning objectives of our courses are critical, what can be sacrificed, and what level of content coverage is “enough.” At the same time, many of our colleagues are, for the first time, becoming painfully aware of some of the rampant inequities in higher education, along several axes of diversity (e.g., socioeconomic disparities mean some students don’t have reliable internet access, older students may have the added burden of caring for young children or elderly parents). We are also trying new things, such as hybrid course design and remote instruction, with varied success.

Prior to COVID-19, instructors had been grappling with how best to encouraging students to contribute to class discussions [3–6]—particularly in the large lecture courses that play a prominent role in undergraduate science education [7–9]. Class participation is a focus of active-learning pedagogy [10], which itself involves a suite of classroom behaviors that involve students in identifying and constructing their own knowledge. Compelling evidence suggests that active learning improves performance and retention outcomes [11], especially for students traditionally underrepresented in STEM disciplines on the basis of their ethnic background, gender, and family history in higher education [12]. However, barriers to *participation* may be particularly acute for students who are underrepresented in science. These disparate patterns of course participation have been posited as both a cause and a consequence of inequities in science higher education based on sociodemographic and affective factors [7, 13–17] and they may have been exacerbated during the recent shift to remote instruction [18, 19]. In a recent annual meeting of the Equity and Diversity in Undergraduate STEM (EDU-STEM; [20]) Network, we—the authors of this work—came to a realization during one of our breakout sessions: while we differed on our sentiments about many aspects of our remote-instruction experience, we all loved the text-based chatting that accompanied our synchronous sessions (via Zoom, Google Meet, Teams, or Blackboard Collaborate). In particular, many of us noticed that the chat feature seemed to encourage more frequent and widespread class participation than we were used to seeing in our in-person lecture courses. This observation piqued our interest and led to the work described here.

Class participation is correlated with a range of positive academic outcomes such as better performance and greater persistence [21, 22]. Situated learning theory provides a framework for understanding the roots of these positive outcomes. Namely, this perspective proposes that learning is a process that occurs through guided, or scaffolded, participation in academic communities of practice [23; see also 24]. In the case of undergraduate science courses, for example, course participation provides a way for students to practice scientific discourse and actively engage in the learning process under the guidance of their instructor. In addition to maximizing learning, participating in academic communities of practice can help students develop a stronger academic identity and, relatedly, a deeper sense of belongingness in academic contexts [25–27]. These outcomes may be especially valuable for students who have historically been underrepresented in STEM fields.

For these reasons, identifying ways to encourage heightened participation among students from all backgrounds has emerged as a key priority in the science education literature [8, 28]. This brings us to the overarching question that motivated this work: *Are synchronous chats a silver lining of emergency remote instruction*? To address this question, we conducted a study of one non-majors introductory biology course with laboratory, offered during Fall 2020. We focus here on the experiences, chat sessions, and perceptions of the instructor (SC) and students in two sections (*n* = 119 and *n* = 118) of this course. Through a survey of student perceptions and an analysis of chats of a dozen class sessions from one of the course sections, we sought to answer the following specific questions:

- How do students use the chats feature in zoom (e.g., course business, content-related question, content-related comment) during synchronous, online course sessions?
- Who is chatting? How frequently and equitably do students participate using the chats feature?
- What affordances or challenges do students perceive from the synchronous chats? Do perceptions of challenges and affordances vary as a function of students’ race/ethnicity, family history in college, or gender?
- How can instructors carry forward the positive aspects of zoom chatting when they return to face-to-face instruction?

## Methods

The University of Minnesota IRB determined this work exempt from full review, due to the low-risk nature of the investigation. Consent to participate in this research was obtained from all students involved.

We analyzed student behavior and perceptions of synchronous chats in a convenience sample of undergraduates enrolled in an introductory biology course. This course, The Evolution and Biology of Sex, is designed as an introduction to biological principles and scientific processes, all through the lens of sex—sexual reproduction, sex and gender, sexual orientation, and sexual life histories. This is a large-enrollment course, specifically for individuals not majoring in biology, and enrolls students from a broad array of disciplines (e.g., the arts, humanities, business). The course is offered in multiple sections, but we focus here on the experiences of the students and instructor (SC) of two sections (*n* = 119 and *n* = 118) offered in Fall 2020. The course was set up as a mix of asynchronous (pre-recorded mini-lectures and readings) and synchronous (class discussions, involving in-class polling, verbal contributions, and text-based chatting via Zoom) elements. The synchronous sessions met twice each week, for 15 weeks, 75 minutes each session.

During the first class session, the instructor established the chat function as a legitimate and welcome mode of communication, by asking students to simply share their major field of study. This input was used to demonstrate the diversity of academic pursuits represented in the class, to confirm that they were not surrounded (virtually) by science majors who may know a lot of biology already, and to set the stage for the use of the chat function. Chats were used in each subsequent class session, either informally (e.g., students raising and often answering questions about the material) or formally (e.g., the instructor asking for a certain number of students to suggest answers to a question posed that day). Simply put, the chat function was enthusiastically integrated into the synchronous class sessions.

### Dissecting the chats

All class sessions were recorded and shared with the students. With student consent, the text-based chatting (hereafter the “chats”) was summarized and then qualitatively analyzed alongside the media (keynote slides) and verbal dialogue. Chat summaries include the number of individual texts contributed during each class session by both students (student total) and the instructor (instructor total), along with the number of individual students that participated in the chats (individual students). Although we had student names, we did not attempt to assign gender or any other descriptors of student identity to those contributing to the chats.

To analyze the chat data, we conducted a content analysis of chat comments. Our coding approach was inductive (i.e., data-driven) in that we were not guided by *a priori* hypotheses during the coding process [see 29]. The decision to use an inductive approach was informed by the exploratory nature of the current research and, relatedly, the lack of prior research focusing on synchronous chat data. At the outset of the coding process, two of the study authors (KL and SC) met with two undergraduate research assistants to assign codes to individual comments. In line with our inductive approach to data analysis, the research assistants were not provided with coding instructions beyond “let’s describe what is actually happening during these chats.” Although the research assistants were not students in the courses that were analyzed, their status as undergraduate “insiders” suggests that they were particularly well positioned to interpret content in the chats, thus improving study trustworthiness [see 30].

**Fig 1:**
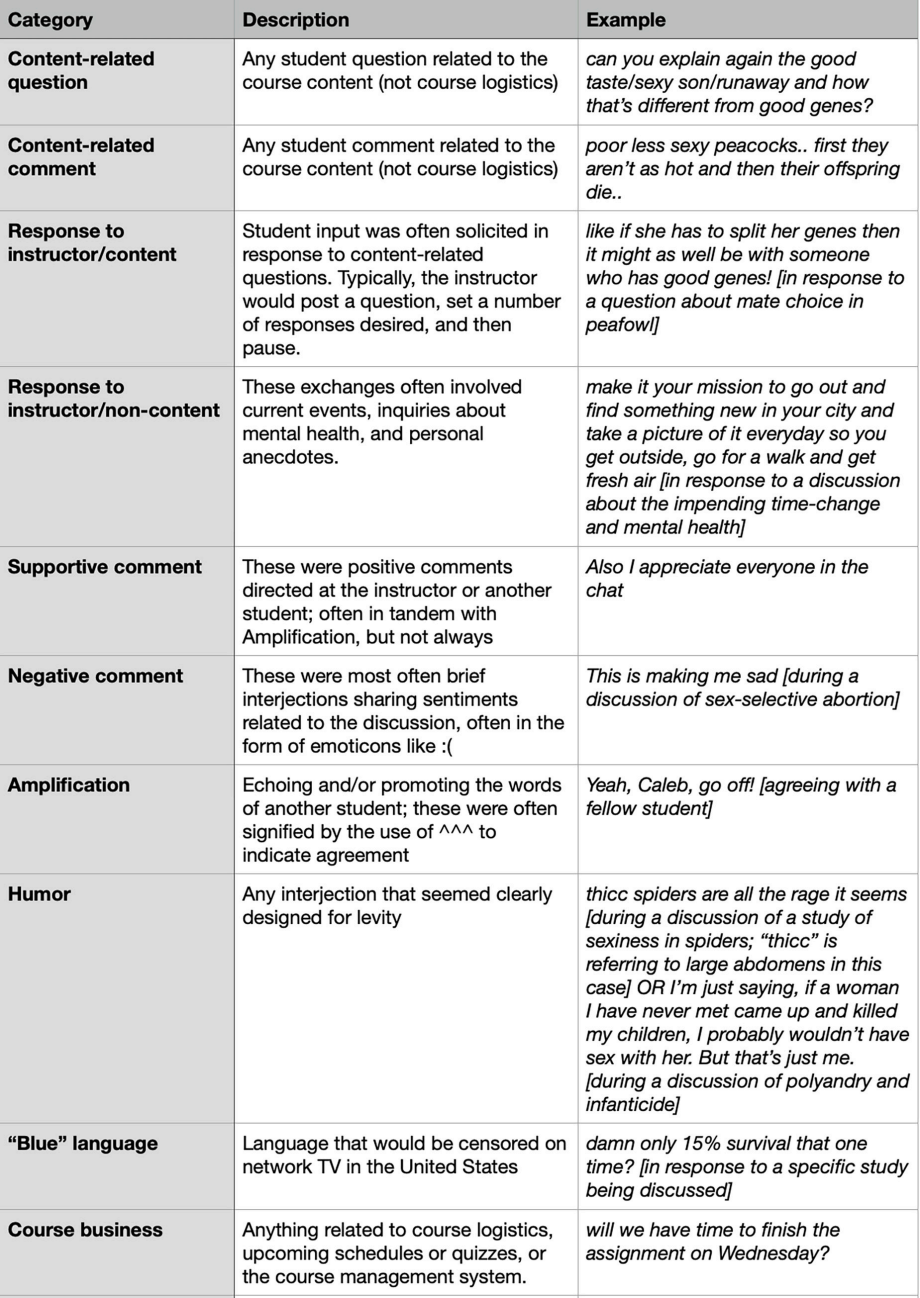
Categories assigned to coded comments, along with an example for each category.

**Fig 2:**
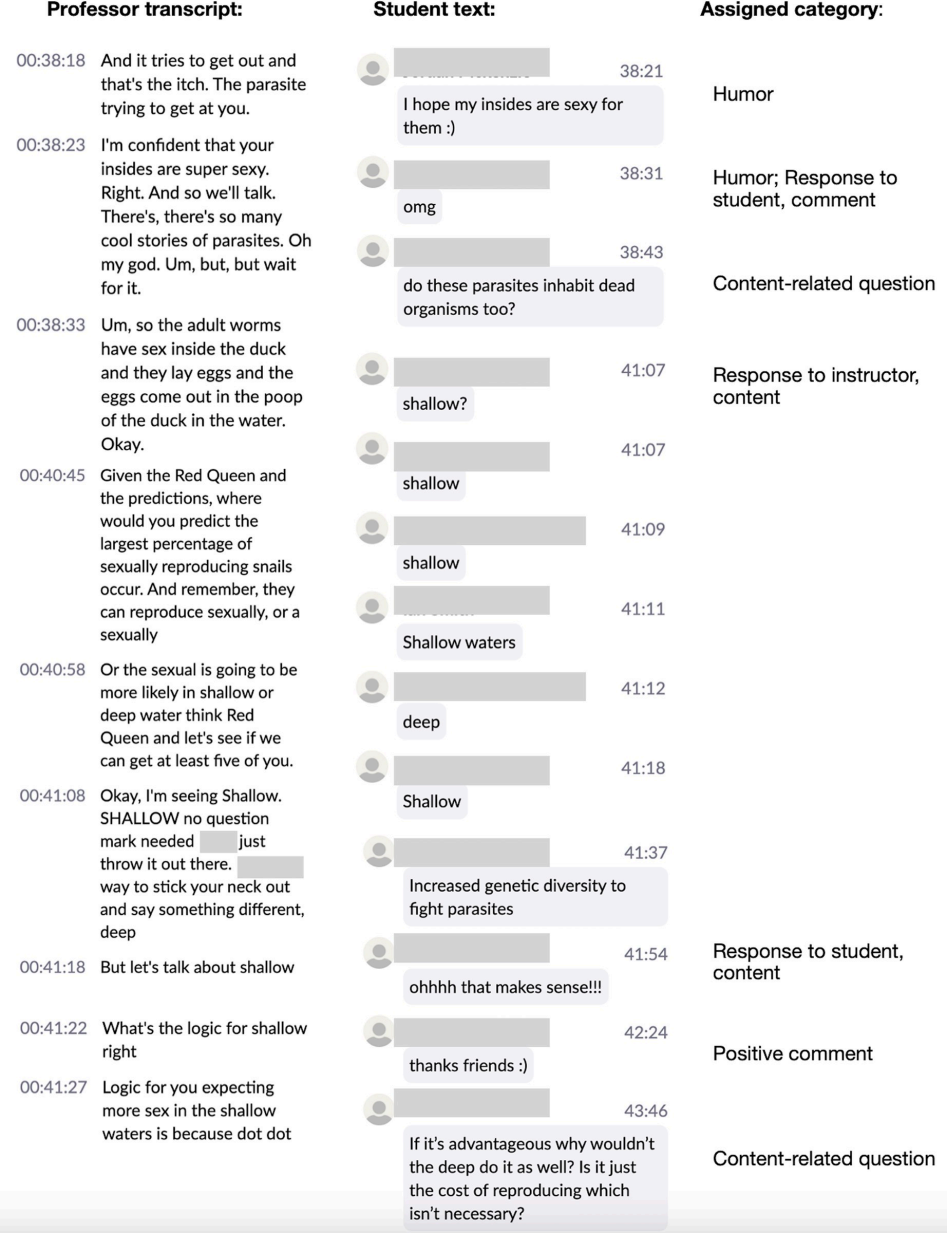
An example of student chatting, during a discussion of mate choice in peafowl, alongside instructor dialogue and how each student chat was coded.

During the initial coding meeting, the research assistants worked with the aforementioned study authors to dissect the comments from one class session (November 4th) and develop a coding scheme. Next, the two research assistants assigned codes to twelve, 75-minute class sessions that occurred during the middle of the semester (from September 21 through October 28). The students then met separately two additional times to come to a consensus agreement on coding. Although intercoder reliability was not assessed, subsequent email communication between the students and SC conveyed that they were overwhelmingly in agreement prior to the consensus meeting. As detailed in Fig 1, the coding process yielded the following themes: content-related question, content-related comment, response to instructor/content, response to instructor/non-content, response to student/content, response to student/non-content, supportive comment, negative comment, amplification (echoing and/or promoting the words of another student), instructor comment, humor, “blue” language (e.g., fuck, damn, dammit), course business. It was common for a single student contribution to be assigned multiple codes. Fig 2 illustrates how a short chat sequence, from one class session, was interpreted using this coding scheme.

### Surveying the students

To build on our analysis of the chat data, we designed a stand-alone survey to gauge student perceptions of synchronous chatting. After all, it would be ill advised to recommend incorporating the chat interface into future courses if students tend to perceive it as ineffective in encouraging their participation or aversive at a more general level. To this end, we developed a series of constrained-choice (Likert scale) items and open-ended items to attain a deeper understanding of students’ experiences with synchronous chatting. We also asked students to report their sociodemographic background, which allowed us to examine whether their perceptions of synchronous chatting systematically varied on the basis of their gender, ethnicity, or family history in college (i.e., whether the student was first-generation or continuing-generation). The final survey was shared in Qualtrics, via the course-management system (Canvas) during the week of November 16^th^ 2020; students were given one week to complete the survey. All student respondents consented to have their anonymized input used for research purposes, using a survey protocol determined to be exempt from full review (due to its low-risk nature) by the University of Minnesota’s IRB.

Roughly 50% of the students enrolled in the course (*N* = 109) completed the survey. Of these students, nearly two-thirds identified as women (63%); the rest identified as men (32%) or reported their gender identity as gender fluid or transgender (5%). We report on binary gender differences to protect the privacy of individuals who identify as gender fluid or transgender. Most participants described their ethnic background as European American; the next-largest ethnic group was African American (6%). In the analyses, we contrasted between students whose ethnicity is historically well represented in higher education (i.e., Asian American or European American; 85%) and students who have historically been marginalized in higher education (i.e., African American; Hawaiian Native/Pacific Islander, or Latinx; 15%). Finally, most students (81%) reported that they were continuing-generation college students (i.e., at least one parent had attended college).

## Results and discussion

*i. How do students use the chat feature in Zoom*?

**Fig 3:**
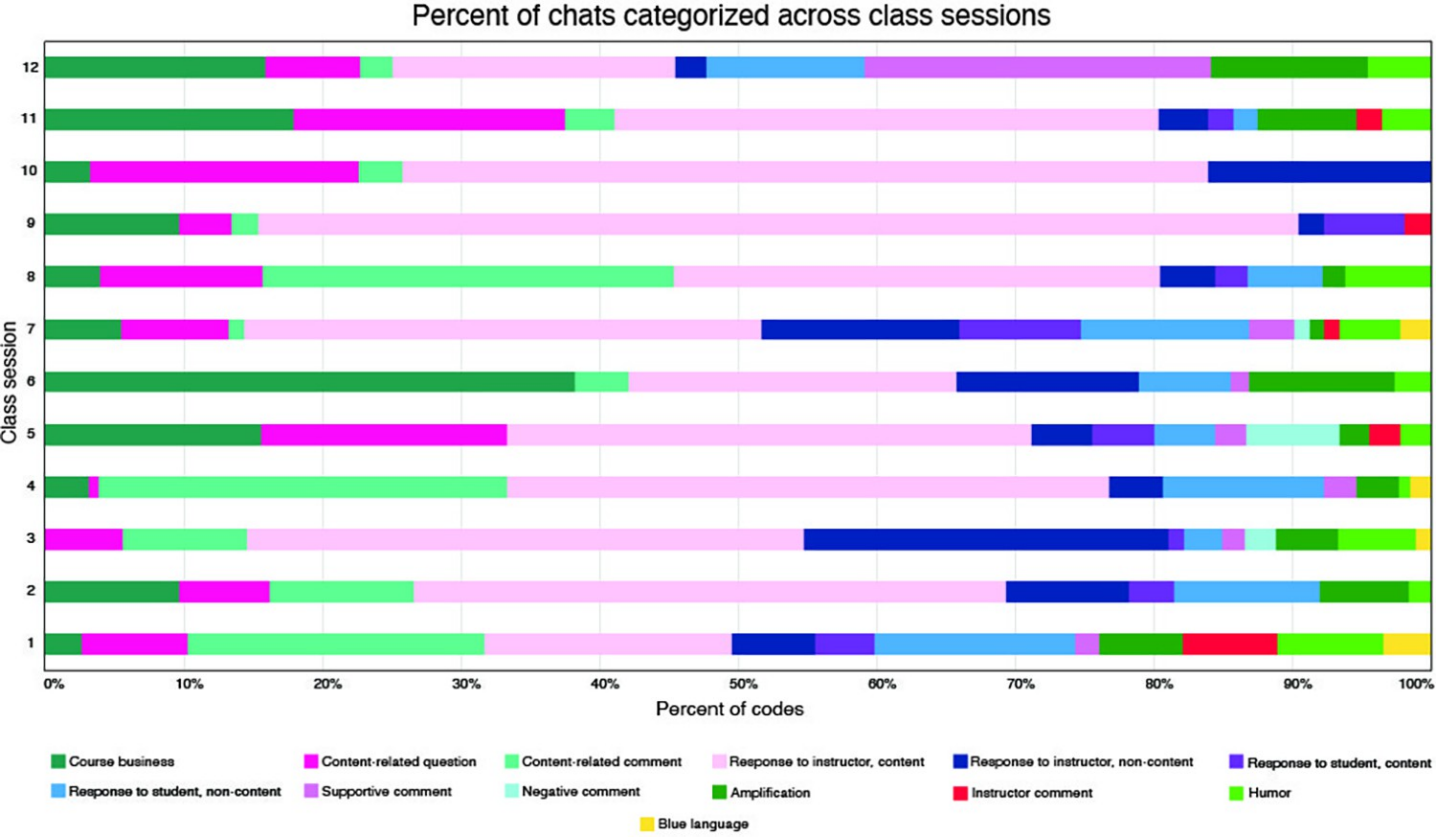
The results of a “dissection” of the chatting in twelve class sessions. Percentages are based on total codes, rather than total texts, thus they sum to 100% for each course. Note that many individual texts were assigned to multiple categories.

The most common code assigned to student texts during these chats was, overwhelmingly, response to instructor/content, with an average of 39 texts per class session being assigned to this code. These were followed by course business (10 texts per class, on average), content-related comment (10), and content-related question (9). At the other end of the spectrum, blue language and negative comments averaged less than 1% of the chat dialogue. Fig 3 depicts the nature of the chats for these twelve class sessions.

Given that content-related chatting characterized three of the top four codes, on average, used in synchronous chatting, it is clear that the majority of the student contributions relate to course content. Also, with negative comments averaging less than 1% of the total texts per class, and supportive comments and amplification averaging 3% and 5%, respectively, of the total, it seems that the texts are more likely to have been supportive of their peers than otherwise. Other findings may require elaboration; for example, in class session six (Fig 3), there was a long series (29, out of 90 total) of chats in which students were doing some “venting” about confusion with a recent update to the course-management software. On days (i.e., one, four, and eight) with more course-related comments, the material was among the most provocative and engaging (e.g., the costs of sexual reproduction, the evolution of mating preferences, gender bias in teaching evaluations, respectively) and elicited more input from students than, e.g., protein synthesis on day twelve. On day nine, the large proportion of “response to instructor, comment” codes reflects the intentional use of the chat during in-class Punnett square exercises, whereby students were prompted to provide gamete genotypes and predicted offspring phenotypes during some short case studies.

*ii. Who is chatting*? *How frequently and equitably do students participate using the chats feature*?

Daily attendance during the semester ranged from ~70% to >95% of the enrollment, in both sections. Thus, on any given day, there were between 80 and 107 students logging in to the Zoom session. For the twelve class sessions examined for this study, daily attendance ranged from 86 to 104 students. The number of individual students participating in the chats ranged from 17 to 32, or between ~15% and ~30% of those in attendance. The number of individual texts ranged from 31 to 160. The instructor contributed between zero and eight texts, but most (seven of the 12) days didn’t text at all.

By these metrics of participation—attendance, number of students chatting, and number of individual texts submitted to the chat—the remote sessions surpassed participation in traditional, in-person classes for this course. For example, in a prior study involving an in-person, pre-pandemic section of this class [8], total student interactions averaged 17.5 per session (with 16 classes observed). This is, admittedly, an unfair comparison in several ways. For students with reliable Wi-Fi access, attendance in a Zoom session is almost effortless—they are free of any transportation (time, money, parking) logistics in attending class, and can attend class without even getting out of bed (which we suspect was the case with many individuals). In response to an instructor question, a dozen students can submit something in the chat synchronously, leading to minimal disruption or lag time as they await being called on by the instructor. But these caveats to comparison constitute the very reasons that chatting lowers barriers to participation, discussed below.

*iii. What affordances or challenges do students perceive from the synchronous chats*?

To better understand students’ subjective perceptions of synchronous chats, we asked them to rate the extent to which the chat feature provided various affordances and challenges. Specifically, students rated their agreement with statements such as *The live chat feature helps me feel more comfortable participating in class discussions* (an affordance) and *Students are disrespectful to each other in live chat* (a challenge). All statements were rated on a 7-point scale ranging from 1 (*disagree strongly*) to 7 (*agree strongly*). Table 1 provides students’ mean ratings for each of the affordances and challenges included on the survey.

**Table 1:** One-sample t-test comparing student ratings against the neutral midpoint on the response scale.

	*M*	*SD*	*t*	*p*
**Affordances**				
1. The professor appreciates the comments I make on live chat.	6.12	1.16	18.60	< .001
2. Live chat makes this class fun.	6.06	1.39	15.33	< .001
3. I enjoy reading the comments other students make in live chat.	5.83	1.53	12.49	< .001
4. Live chat helps me stay engaged during class.	5.80	1.59	11.76	< .001
5. Live chat helps me learn.	5.69	1.49	11.78	< .001
6. Live chat helps me feel more comfortable participating in class discussions.	5.65	1.61	10.65	< .001
7. Live chat helps me feel connected to other students.	5.56	1.68	9.64	< .001
8. Other students appreciate the comments I make in live chat.	5.07	1.23	8.83	< .001
**Challenges**				
1. Other students have said things in live chat that make me uncomfortable.	2.74	1.92	-6.813	< .001
2. Students are disrespectful to each other in live chat.	2.47	1.56	-10.17	< .001
3. Other students have made me feel embarrassed about something I said in live chat.	1.87	1.36	-16.08	< .001
4. The professor has made me feel embarrassed about something I said in live chat.	1.52	1.15	-22.01	< .001

**Note.** Ratings were made on a scale that ranged from 1 (*disagree strongly*) to 7 (*agree strongly*). The critical value for the 1-sample *t*-test was 4 (*neither agree nor disagree*).

When testing the student means against the scale midpoint to gauge endorsement of each affordance and challenge (see Table 1), Bonferroni-corrected *p*-values were used to counteract the likelihood of Type I error. More specifically, the critical value for assessing the statistical significance was .006 for the eight affordances and .01 for the four challenges. As explained earlier, we also examined whether perceptions of the chat function systematically varied as a function of student background (i.e., ethnicity, family history in college, and gender); however, only the analyses pertaining to gender were significant. We elaborate on these findings below.

### Affordances

Mean ratings for all seven affordances were significantly higher than the scale midpoint, which was neutral in valence (see Table 1). That is, on average, students agreed that the live chat feature granted each of the affordances we included on the survey. Below, we elaborate on two affordances of particular interest. Our quantitative analysis is supplemented with qualitative data that students provided in response to open-ended questions about remote learning and synchronous chat.

#### Enjoyment

Survey responses indicated that students tended to enjoy the synchronous chat aspect of class. On average, students agreed with statements such as *The live chat feature makes this class fun* (*M* = 6.06/7) and *I enjoy reading the comments other students make in the chat* (*M* = 5.83/7). Although both women’s and men’s ratings of these two items were significantly higher than the scale midpoint (see Table 1), women’s ratings were significantly higher than men’s (*p*s < .01). Thus, both women and men enjoyed synchronous chat, but women enjoyed it more.

Students’ enjoyment of synchronous chat is also reflected in their open-ended survey responses. When asked whether there were aspects of virtual learning that they enjoyed, a critical mass (*N* = 20) noted that live chat was one of the things they enjoyed most. (Many responses focused on aspects of virtual learning that are tangential to pedagogy and the learning environment such as not having to commute to campus). Importantly, students responded to this question at the beginning of the survey before we had asked any other questions. Thus, they were not primed to focus on the chat feature in their responses. Representative examples of open-ended responses that focus on synchronous chat come from Chloe and Brian (all names are pseudonyms). For example, Chloe appreciated that live chat enables a wider range of students to participate: “I think it is easier for students to speak up (chat) in the online format since they can just type in their inputs instead of having to talk in front of the whole class so I feel like the online experience is allowing me to hear from more students.” Relatedly, Brian explained, “I really like being able to use the chat feature. It is a low stress way to be heard and ask questions in class. I also like how we can interact with other students in this way.”

#### Participation

In addition to enjoying synchronous chat, students also tended to report it helped their course participation. On average, students agreed with the following statement: *Live chat helps me feel more comfortable participating in class discussions* (*M* = 5.65/7). Both women’s and men’s ratings of this item were significantly higher than the scale midpoint (see Table 1); however, women’s ratings were significantly higher than men’s (*p* < .01), which illustrates that women were especially likely to report that live chat enhanced their participation.

These findings align with students’ open-ended data. Of the students who highlighted live chat as a positive aspect of virtual learning, many explicitly noted that live chat makes it easier to participate in class discussions. A representative open-ended response along this vein comes from Jessica, who explained that she participates more in live chat than she would in a traditional in-person lecture:

“Our class lecture is 100+ people but on Zoom, we are all active in the ‘chat.’ In-person I know I would not participate nearly as much since it would require speaking in front of the whole class. I think this Zoom chatting function makes participation for large lectures go up and people feel comfortable to share directly with the class and teaching team.”

We suspect, based on other student comments, that the low-stakes nature of the chats also makes the chat function more appealing to students with certain disabilities. For example, Devi shared that:

“I feel more comfortable being able to type it out so I can make sure what I’m saying makes sense, and I don’t have to worry about stuttering.”

And Crystal echoed the comments of several students who expressed social anxiety in large-class settings, and an appreciation for the ability to chat:

“Sometimes I get anxious about speaking up in class and being able to just type it in reduces some of my anxieties especially in a class this large.”

### Challenges

As noted, the survey also included questions about challenges that synchronous chats might present. Mean ratings for all four challenges were significantly lower than the scale midpoint (Table 1). That is, on average, students disagreed that the live chat feature contributed to the challenges that we included on the survey. The most strongly endorsed challenge was *Other students have said things in live chat that make me uncomfortable* (*M* = 2.74/7). Only one challenge showed a significant gender difference: Relative to women, men were significantly more likely to agree that *The professor made me feel embarrassed about something I said in live chat* (*p* = .02). It merits reiterating, however, that mean ratings of this challenge were quite low among the men in the sample (*M* = 1.81/7).

It was rare for students to highlight challenges specific to synchronous chat in their open-ended responses. The students who did discuss challenges often provided idiosyncratic reflections that were not echoed by others in the class. For example, Matt noted that some students are “keyboard warriors” who comment too frequently, but then went on to explain that the chat is still generally beneficial because it provides an efficient way to ask questions.

While there are multiple avenues for student feedback and engagement around course material, methods to gauge real-time student comprehension and attentiveness are limited. Synchronous chatting allows spontaneous feedback, including the instructor’s ability to modify planned questions and prompts for engagement. In contrast, some polling tools require predetermined instructor prompts, which limit our ability to tailor questioning to the feedback received during the class period. Polls, on the other hand, are typically anonymous, which may address the challenge of students feeling embarrassed or singled out for their answers. Importantly, chats provide a clear avenue for student-student dialogue which is otherwise difficult or impossible to achieve during remote instruction, and which surpass metrics of student-student participation during traditional in-person lecture-based courses.

### Synchronous chat in future classes

When asked whether they would like to see synchronous chat incorporated into their future courses, students tended to agree that this would be worthwhile. On average, students agreed with the following statements: *I think the live chat feature should be included in future classes like this* (*M* = 6.20/7) and *I wish my in-person classes had something like the live chat feature* (*M* = 5.10/7). As with the affordances discussed earlier, women’s mean ratings for both items were significantly higher than men’s mean ratings (*p*s < .01). Importantly, however, another gender difference emerged for the question asking about whether live chat should be incorporated into future in-person courses. Specifically, the mean rating for women was significantly higher than the scale midpoint (*M* = 5.61/7), whereas the mean rating for men was not (*M* = 4.06/7). This means that men tended to have a neutral stance on whether live chat should be incorporated into future in-person courses. In contrast, women tended to be more enthusiastic about this possibility.

In sum, students in the present study tended to enjoy synchronous chatting and, on average, did not perceive many negatives of this mode of participation. Relative to men, women were more likely to note that the chat feature was enjoyable and made them feel more comfortable participating in class discussions. They were also more likely than men to express a desire to utilize live chat in their future in-person courses. These patterns are intriguing given that course participation provides an important active learning opportunity that may enhance belongingness and academic identity [23]. This implies that implementing strategies such as live chat may help to address stubborn gender gaps in STEM degree attainment [31] and help women feel more comfortable in a space that has not always welcomed them [32–34]. It merits noting that perceptions of chat did not differ on the basis of students’ racial-ethnic background or family history in college. These analyses may have been underpowered, but it may also be the case that other strategies are needed to promote class participation among members of these groups.

*iv. How can instructors carry forward the positive aspects of zoom chatting when we return to face-to-face instruction*?

Not only do the above findings suggest that the chats are valued by the students, there are indications that the chats may lower barriers to participation for students less inclined to participate in large-class discussions. Due to low sample sizes, we are unable to extend this claim beyond disparities due to gender, but future work will assess whether students from other underrepresented groups in STEM may benefit from text-based chatting options.

Should these trends persist in broader, more systematic studies of synchronous chatting, it behooves us as educators to consider how to incorporate this dynamic into the face-to-face classroom. Admittedly, one option doesn’t involve the face-to-face environment at all, but rather involves leveraging hybrid instruction modalities to allow for more diverse lines of communication. Ballen et al. [8] found evidence that using a diversity of channels for communication increased the likelihood of in-class participation from women in introductory-biology courses. Hybridizing a course, and including synchronous remote options, is one way to diversify these channels, and allows students to participate in text-based chatting. This is, admittedly, cumbersome, given that many hybrid-course scenarios involve asynchronous—rather than synchronous—online work.

We may also learn from recent work on “backchannels,” tools that allow digital conversations to occur during a class session [35]. Several of these tools have been developed (e.g., *Backstage*, [36]) recently, but in general they allow students to contribute to course dialogue through an app on their devices, with their input projected—typically anonymously—on a shared screen. Some investigators have indicated that, in line with our initial findings, these backchannels can increase the number—and possibly the diversity—of students engaging in class [35, 37–39]. However, research on the utility and impact of these backchannels is still emerging. Moreover, access to these digital resources may be cost-prohibitive if they require an expensive institutional license. Further, some students may not have reliable access to the technology needed to run the applications that correspond to these resources. Accordingly, the benefits of implementing backchannel tools need to be considered alongside the possibility that implementation could widen existing inequities.

## Conclusions

Our findings lead us to draw two key conclusions and recommendations for future research: *Further research is needed to determine which instructional decisions lead to chats that contribute to positive learning environments*. While we demonstrated that text-based chatting encourages participation, future work can identify how best to foster effective in-class communication and what type of chat dialogue is optimal. We hypothesize that student participation in chats is largely predicated on the instructor’s encouragement of, and response to, synchronous chatting. An instructor that ignores the chatting, or even suggests students participate vocally instead, will likely not experience chatting the way we describe here. Ongoing follow-up work on text-based chatting—looking at multiple courses, at multiple institutions, and surveying those students and instructors—will help clarify what factors are associated with constructive student engagement via chatting. Another priority for future research is to examine whether findings from the current study extend to students who are majoring in STEM fields. Given that course participation can be a challenge for students from a range of academic backgrounds and women more generally [8], we suspect that a study focusing on STEM majors would reveal patterns similar to those obtained in the current research; however, this is a tentative prediction that needs to be examined through empirical research in light of work indicating that STEM majors and non-majors differ from one another in meaningful ways STEM majors [40].

*Used appropriately*, *something akin to synchronous chats could provide an avenue for course participation for students less likely to “speak up” verbally in whole-class*, *face-to-face discussions*. **Our study is the first, to our knowledge, to document a stronger preference for the chats option among women, who are less likely than men to participate in large introductory science classrooms** [8, 9]. Given that studies that test the impacts of active learning environments support the benefits of participatory engagement [11], and that gender bias in STEM persists [32–34, 41–43], a critical evaluation of how we encourage or restrict student participation is necessary. Perhaps recent experiences with synchronous chats have given our colleagues inspiration to pursue more options for in-class engagement. Given the recent work presenting some potential perils of speaking up [14, 44], text-based chatting could be part of the solution. In sum, we encourage our colleagues to view text-based chatting as an important element in their toolbox of resources to solicit engagement, from *all* students.
